# Implementing Evidence‐Based, Phase‐Specific Multimodal Analgesia for Total Joint Arthroplasty: An Interprofessional Review for Nursing Practice and Management

**DOI:** 10.1155/jonm/1182729

**Published:** 2026-08-13

**Authors:** Qiuru Wang, Dongmei Zhao, Jian Hu, Changjun Chen, Ting Ma, Xiaoyan Liu, Jing Yang, Pengde Kang

**Affiliations:** ^1^ Department of Orthopedic Surgery, West China Hospital of Sichuan University, 37# Wainan Guoxue Road, Chengdu 610041, China, wchscu.cn; ^2^ Department of Orthopedic Nursing, West China Hospital, Sichuan University, 37# Wainan Guoxue Road, Chengdu 610041, China, scu.edu.cn; ^3^ Sports Medicine Center and Department of Orthopedics and Orthopedic Research Institute, West China Hospital, Sichuan University, 363# Furong Avenue, Chengdu 610041, China, scu.edu.cn; ^4^ Department of Anesthesiology, West China Hospital of Sichuan University, 37# Wainan Guoxue Road, Chengdu 610041, China, wchscu.cn; ^5^ Department of Orthopedic Surgery, Shandong Provincial Qianfoshan Hospital, 16766# Jingshi Road, Jinan 250000, China, sdhospital.com.cn; ^6^ Department of Perioperative Nursing, Anesthesia and Surgery Center, West China Hospital, Sichuan University, 37# Wainan Guoxue Road, Chengdu 610041, China, scu.edu.cn

**Keywords:** enhanced recovery after surgery, multimodal analgesia, nursing management, pain, total hip arthroplasty, total knee arthroplasty

## Abstract

**Purpose:**

This review aims to synthesize current evidence on perioperative multimodal analgesia within enhanced recovery after surgery (ERAS) pathways for total joint arthroplasty (TJA). The primary objective is to translate this evidence into clear, actionable insights for nursing management and interprofessional collaboration, emphasizing the pivotal role of nursing professionals in orchestrating patient‐centered pain management to optimize outcomes.

**Methods:**

A structured narrative review was conducted to provide a clinically relevant synthesis of current evidence on perioperative multimodal analgesia within ERAS pathways for TJA. The review methodology was informed by established recommendations for rigorous narrative reviews, including the SANRA framework. PubMed and MEDLINE were searched for English‐language publications available up to June 2025. Studies were screened for relevance and prioritized according to methodological quality, clinical significance, and implications for nursing management and interprofessional practice, with particular emphasis on randomized controlled trials, cohort studies, clinical practice guidelines, systematic reviews, meta‐analyses, and landmark studies.

**Results:**

Multimodal analgesia, which synergistically combines pharmacologic and nonpharmacologic techniques, is the standard of care for TJA. Evidence supports phase‐specific protocols: preemptively, combining analgesics (e.g., COX‐2 inhibitors) 24–48 h preoperatively and tailoring regimens to patient risk profiles (e.g., duloxetine for central sensitization); intraoperatively, using local infiltration analgesia (LIA) as a cornerstone technique; and postoperatively, implementing a structured, nursing‐led “non‐opioid‐first” strategy with scheduled NSAIDs/acetaminophen and monitored opioid rescue protocols. For total knee arthroplasty (TKA), the combination of adductor canal block (ACB), IPACK block, and LIA is an evidence‐based strategy for optimizing analgesia while preserving motor function. Intravenous glucocorticoids are also recommended during surgery. Successful implementation is critically dependent on nursing management, including proactive patient education and expectation management, vigilant interpretive pain assessment, systematic monitoring for medication‐specific adverse effects, and coordination of mobilization with analgesic peaks. The heterogeneity of evidence underscores the need for standardized, institution‐specific protocols, implementation tools, and governance frameworks to support nursing‐led ERAS practice. Ultimately, effective analgesia functions within an interprofessional collaborative framework, in which nurses act as central coordinators facilitating communication among surgeons, anesthesiologists, and therapists to dynamically personalize care.

**Conclusions:**

The evolution toward personalized multimodal analgesia elevates the nursing role from protocol administration to essential clinical decision‐making and care coordination. Nursing leadership is indispensable for bridging evidence‐to‐practice gaps, minimizing practice variation, and ensuring patient safety. Key to success is the adoption of standardized pathways that empower nurses to lead patient education, assessment, and cross‐disciplinary communication. Future efforts should focus not only on refining analgesic evidence but also on implementation science, protocol governance, workforce competency development, and quality improvement strategies that facilitate sustainable integration into nursing workflows and consistently achieve superior analgesia, enhanced functional recovery, and minimized complications within ERAS programs.

## 1. Introduction

Total joint arthroplasty (TJA), including total knee arthroplasty (TKA) and total hip arthroplasty (THA), has become an effective intervention for end‐stage knee and hip diseases, significantly alleviating patient pain and improving function [1, 2]. Data from the United States demonstrate that its demand remains resilient to economic fluctuations. For instance, during the economic downturn in 2009–2010, the volume of primary THA and TKA still increased by approximately 6%, while revision surgeries experienced even greater growth—hip revisions by 10.8% and knee revisions by 13.5%. This reflects its solid clinical value and fundamental patient need [3]. Projections based on demographic changes in the U.S. population and national health expenditure models indicate that the volume of TJA will continue to increase into 2021 and beyond, with the projected growth for THA potentially exceeding previous model predictions [4].

Enhanced recovery after surgery (ERAS) protocols emphasize early postoperative mobilization, reduced hospital stays, and improved patient satisfaction while ensuring safety [5]. The successful implementation of ERAS in TJA is inherently multidisciplinary, requiring seamless collaboration across surgical, anesthetic, and nursing teams to optimize the entire care pathway [6–8]. Within this framework, nursing professionals play a pivotal role in executing and coordinating evidence‐based strategies, including pain management, which is critical to achieving rapid recovery goals.

Over the past 2 decades, perioperative management for TJA based on ERAS principles has achieved significant progress. However, postoperative pain management remains a central challenge for the entire care team, with nursing at the forefront of assessment, monitoring, and intervention [9, 10]. TJA is among the most painful surgical procedures [11–13]. It has been reported that more than 60% of patients experience severe pain following TKA [11, 12], while a majority of patients report significant pain after THA [13]. Postoperative pain ranks among the top concerns for TJA patients [9]. Such pain not only causes patient suffering but also creates significant management complexities, including barriers to early mobilization, increased risk of complications (e.g., thromboembolism, prolonged opioid use), and heightened demands on nursing vigilance and multidisciplinary coordination to support functional recovery [14–16].

**FIGURE 1:**
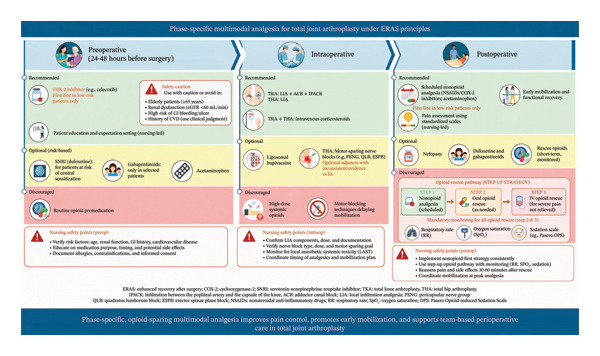
Phase‐specific multimodal analgesia for total joint arthroplasty under enhanced recovery after surgery principles. Abbreviations: ERAS, enhanced recovery after surgery; TJA, total joint arthroplasty; TKA, total knee arthroplasty; THA, total hip arthroplasty; LIA, local infiltration analgesia; ACB, adductor canal block; IPACK, infiltration between the popliteal artery and capsule of the knee; COX‐2, cyclooxygenase‐2; SNRI, serotonin–norepinephrine reuptake inhibitor; GI, gastrointestinal; CVD, cardiovascular disease; eGFR, estimated glomerular filtration rate; PENG, pericapsular nerve group block; QLB, quadratus lumborum block; ESPB, erector spinae plane block; NSAIDs, nonsteroidal anti‐inflammatory drugs; IV, intravenous; RR, respiratory rate; SpO_2_, peripheral oxygen saturation; OPS, Opioid‐induced Sedation Scale; LAST, local anesthetic systemic toxicity.

With the continuous advancement of surgical and analgesic techniques, perioperative analgesia strategies for TJA have also continuously evolved, necessitating a reassessment of current evidence. The concept of multimodal analgesia is now widely adopted for postoperative pain management in TJA. Nevertheless, substantial variations persist in specific analgesic approaches across different regions in both clinical practice and research, posing challenges for standardizing nursing practices and interprofessional workflows. A critical review of existing pain management strategies is essential for understanding their practical implications for bedside nursing, including advantages, limitations, and applicable scenarios. This facilitates the development of clinically actionable, personalized perioperative analgesia protocols that nursing teams can effectively implement. The purpose of this review is therefore to summarize current research advances in ERAS‐based perioperative pain management for TJA and, crucially, to translate this evidence into clear insights for nursing management and interprofessional collaboration. Figure 1 summarizes the key concepts and findings organized in this review.

## 2. Literature Search Strategy

This review was conducted as a structured narrative review designed to provide a comprehensive and clinically relevant synthesis of current evidence regarding ERAS‐based perioperative pain management for TJA, with particular emphasis on implications for nursing management and interprofessional practice. The review methodology was informed by published recommendations for rigorous narrative reviews, including the guidance proposed by Sukhera and the Scale for the Assessment of Narrative Review Articles (SANRA) framework developed by Baethge et al. [17, 18].

To identify relevant literature, a structured search was performed in PubMed and MEDLINE for English‐language publications available up to June 2025. Search terms included combinations of keywords related to ERAS, TJA, TKA, total hip arthroplasty, multimodal analgesia, perioperative pain management, opioid‐sparing strategies, regional anesthesia, and local infiltration analgesia (LIA).

Consistent with the objectives of a narrative review, studies were not selected through a formal systematic review process. Instead, articles were screened for relevance to the review topic and prioritized according to methodological quality, clinical significance, and relevance to perioperative multimodal analgesia and nursing practice. Particular emphasis was placed on randomized controlled trials, cohort studies, clinical practice guidelines, systematic reviews, meta‐analyses, and landmark studies reporting outcomes, such as postoperative pain, opioid consumption, functional recovery, complications, and implementation considerations. Duplicate publications, case reports, editorials, conference abstracts, and non‐peer‐reviewed publications were excluded.

The selected literature was critically reviewed and synthesized to summarize current evidence, identify areas of consensus and controversy, and highlight implications for nursing management, interprofessional collaboration, and future research within ERAS pathways for TJA.

## 3. Multimodal Analgesia

Multimodal analgesia is a strategy that synergistically targets pain transmission pathways by combining medications with different mechanisms of action (e.g., nonsteroidal anti‐inflammatory drugs [NSAIDs], acetaminophen, local anesthetics, and opioids) and nonpharmacologic techniques (e.g., peripheral nerve blocks and LIA). This approach enhances analgesic efficacy while reducing side effects associated with monotherapy, such as opioid dependency and nausea [19, 20]. Its successful implementation in perioperative care for TJA—including TKA and THA—is now the clinical standard and fundamentally relies on coordinated, multidisciplinary execution, with nursing professionals playing a central role in its daily orchestration at the bedside [21, 22].

In practice, this strategy is operationalized through a phased approach that requires proactive nursing management: preoperative preventive multimodal analgesia, intraoperative multimodal analgesia, and postoperative multimodal analgesia. Extensive evidence demonstrates that effectively delivered multimodal analgesia significantly mitigates acute pain, facilitates early functional exercise, shortens hospital stays, and reduces the incidence of postoperative chronic pain and other complications [21–24]. Achieving these outcomes, however, depends heavily on consistent nursing interventions, such as vigilant pain assessment, timely administration of scheduled medications, monitoring for treatment responses and adverse effects, and close collaboration with physiotherapists and physicians to align analgesia with mobilization goals [25].

Therefore, multimodal analgesia is not merely a pharmacological concept but a foundational framework for nursing‐led, patient‐centered pain management within the ERAS pathway.

## 4. Preemptive Multimodal Analgesia

As a foundational component of the ERAS pathway, preemptive multimodal analgesia is initiated before surgery to proactively manage pain and requires careful preparation and patient education led by the nursing team [26]. Its core principle lies in preemptively blocking or attenuating the transmission of noxious stimuli to the central nervous system by administering a combination of analgesics with distinct mechanisms of action, before actual tissue damage caused by surgery. For nursing practice, this translates to ensuring timely administration, monitoring for early effects, and educating patients on the rationale behind “preventing pain before it starts” [27].

Preemptive multimodal analgesia typically involves a combination of orally administered analgesics with distinct mechanisms alongside peripheral nerve blocks [27, 28]. Common preoperative oral regimens include selective COX‐2 inhibitors, extended‐release opioids, gabapentinoids (e.g., gabapentin and pregabalin), acetaminophen, and duloxetine for patients with central sensitization [29, 30]. Nursing responsibilities are critical here: verifying medication history (especially opioid tolerance), administering pre‐admission medications as ordered, and initiating monitoring for specific drug‐related side effects (e.g., dizziness with gabapentinoids and gastrointestinal symptoms with NSAIDs) [31]. No gold standard exists for preventive analgesic oral combinations. Selective COX‐2 inhibitors are widely regarded as the cornerstone. Clinicians typically build drug combinations around selective COX‐2 inhibitors (e.g., celecoxib), supplementing them with other agents, a process where clear nursing‐physician communication regarding the regimen’s purpose and monitoring plan is essential [28, 32].

Recent Evidence and Implications for Nursing Monitoring:

1. Extended‐Release Opioids: Recent evidence challenges their efficacy, showing they may worsen outcomes for opioid‐naïve patients [27, 30]. This underscores the nursing imperative to identify patients with pre‐existing opioid use during pre‐admission assessment and flag them to the team, as their analgesic plan will differ significantly.
2. Gabapentinoids: Evidence is mixed. While guidelines note limited benefit for gabapentin, pregabalin may reduce opioid needs, and combining it with a COX‐2 inhibitor shows promise [26, 33].
3. Serotonin–Norepinephrine Reuptake Inhibitor: Duloxetine is specifically indicated for patients screened for central sensitization [34]. Nursing must be aware of the specific indications for these drugs and monitor for neuropsychiatric side effects (somnolence, dizziness) that could impact fall risk during early mobilization.
4. Acetaminophen: Evidence for added benefit in preemptive regimens is limited [35]. Nursing’s role is to ensure safe administration within daily dose limits, particularly if continued postoperatively.

Due to insufficient high‐quality studies examining the specific contributions of individual agents within heterogeneous baseline drug combinations, substantial research is needed to establish optimal protocols [27–30, 32, 33, 35]. This variability in practice highlights the importance of unit‐based protocols and standardized nursing checklists to ensure safe and consistent preemptive analgesic administration.

Currently, no gold standard exists for the optimal initiation timing of preemptive analgesia [36]. A recent RCT demonstrated superior analgesia when initiated 24–48 h preoperatively versus 1 h before [28]. From a nursing workflow perspective, initiating 24 h preoperatively is often more practical, allowing for comprehensive patient education, medication reconciliation, and coordination with pre‐admission services. Nurses play a key role in coordinating this timing and reinforcing education during the pre‐admission visit.

The latest key literature on preemptive multimodal analgesia is summarized in Table 1.

**TABLE 1:** Literature summary on preemptive multimodal analgesia.

Authors and year	Reference number	Study type	Sample size	Surgery type	Analgesic techniques discussed	Main results
Shen et al. (2009)	[23]	Randomized controlled trial	60	TKA	Preemptive celecoxib	Perioperative administration of the selective celecoxib holds the effect of preemptive analgesia
Zhou et al. (2023)	[26]	Randomized controlled trial	149	TKA	Preemptive pregabalin combined with celecoxib	Pregabalin combined with celecoxib had positive effects on improving acute pain compared with pregabalin or celecoxib alone
Wang et al. (2023)	[27]	Randomized controlled trial	100	TKA	Preemptive oxycodone	Preemptive oxycodone did not provide benefits over placebo
Wang et al. (2024)	[28]	Randomized controlled trial	120	TKA	Preemptive multimodal analgesia initiated at various time points	Compared with preemptive multimodal analgesia starting at 1 h preoperatively, initiating preemptive multimodal analgesia at 24 and 48 h preoperatively provided better postoperative pain relief
Xu et al. (2017)	[29]	Randomized controlled trial	132	TKA	Preemptive celecoxib combined with low‐dose tramadol/acetaminophen	Preemptive celecoxib combined with low‐dose tramadol/acetaminophen is an effective preemptive analgesia regimen
Cooper et al. (2019)	[30]	Retrospective cohort study	550	TKA and THA	Preemptive oxycodone	Patients who were given preemptive opioids experienced more pain and consumed more postoperative opioids
Passias et al. (2023)	[32]	Retrospective cohort study	1691	TKA and THA	Preemptive three‐medication analgesic therapy (acetaminophen, celecoxib, and gabapentin)	Preemptive three‐medication analgesic therapy can provide better postoperative analgesic effects compared to not using medication
Hannon et al. (2020)	[33]	Clinical practice guidelines	/	TKA and THA	Preemptive gabapentinoids	Gabapentinoids do not reduce postoperative pain, but pregabalin reduces opioid consumption
Wang et al. (2023)	[35]	Randomized controlled trial	80	TKA	Preemptive acetaminophen	Preemptive acetaminophen did not provide benefits over placebo

Abbreviations: h, hour(s); THA, total hip arthroplasty; TKA, total knee arthroplasty.

## 5. Peripheral Nerve Blocks

Peripheral nerve blocks constitute an integral component of multimodal perioperative analgesia for TJA [37–39]. While performed by anesthesiologists, their success is enhanced by coordinated perioperative nursing care, including patient education about the block’s purpose and expected sensations, and postprocedure monitoring for both efficacy and potential complications. No gold standard exists for their optimal administration timing. Given that anesthesiologists typically perform peripheral nerve blocks preoperatively in contemporary clinical practice [37–40], and considering a recent randomized controlled trial demonstrating that preoperative adductor canal block (ACB) in TKA patients—compared to postoperative ACB—reduced in‐hospital opioid consumption, decreased intraoperative and postoperative stress responses, enhanced in‐hospital pain relief, and lowered the incidence of chronic pain at 3 months postoperatively [41], we categorize peripheral nerve blocks within preoperative preemptive multimodal analgesia protocols.

### 5.1. Peripheral Nerve Blocks for TKA

Peripheral nerve blocks initially commonly used for post‐TKA analgesia included femoral nerve block, sciatic nerve block, and ACB [42–44]. While traditional femoral and sciatic nerve blocks provide effective analgesia, they reduce quadriceps muscle strength, impede early postoperative functional exercise, and increase fall risk [45–47]. This necessitates vigilant nursing assessment of motor function and fall risk when these blocks are used. Consequently, in institutions with rapid technological adoption, femoral and sciatic nerve blocks are now rarely employed in ERAS protocols for TKA. The ACB targets the saphenous nerve (sensory nerve), medial femoral cutaneous nerve, and posterior branch of the obturator nerve. This technique has been demonstrated to achieve analgesia comparable to femoral nerve blocks while preserving quadriceps strength [45–48]. Substantial evidence indicates that TKA patients receiving ACB exhibit superior mobility compared to those receiving femoral nerve blocks [45–48]. Nursing plays a key role in assessing and documenting pain relief and motor function post‐ACB to guide safe mobilization with physiotherapy.

However, many patients receiving only an ACB still report severe posterior knee pain, as ACB primarily anesthetizes the anteromedial sensory innervation of the knee and does not cover the posterolateral aspects [49–52]. To address this gap, the ultrasound‐guided infiltration between the popliteal artery and the capsule of the knee (IPACK block) was developed to deliver targeted analgesia to the posterior knee region [53]. Current high‐level evidence including systematic review and meta‐analysis strongly supports a specific multimodal, function‐sparing strategy: the combination of ACB, IPACK block, and LIA. This integrated ACB + IPACK + LIA regimen provides optimal pain relief while maximally preserving quadriceps muscle function and promoting early ambulation, and is widely established as the evidence‐based standard for TKA within ERAS protocols [54–56]. Nursing coordination is vital to schedule postoperative mobilization during peak analgesic effect and to monitor for any breakthrough pain or unexpected motor deficit.

A systematic review and meta‐analysis incorporating 11 randomized controlled trials compared the analgesic efficacy of single‐injection ACB versus continuous catheter ACB [57]. The analysis revealed that continuous catheter ACB failed to enhance or prolong analgesia within 48 h postoperatively compared to single‐injection ACB [57]. Current clinical evidence does not support the routine use of continuous catheter ACB for post‐TKA analgesia. Given its convenience and effectiveness, single‐injection ACB remains the preferred approach. If continuous catheters are used, nursing responsibilities include meticulous site care, monitoring for dislodgement or signs of infection, and managing the infusion pump—key points expanded in the nursing management section. Researchers have also explored supplementing ACB + IPACK + LIA with additional nerve blocks (e.g., obturator nerve block, lateral femoral cutaneous nerve block). However, these adjunctive peripheral nerve blocks demonstrated no significant clinical improvement in postoperative analgesia for TKA [11, 58].

The latest key literature on peripheral nerve blocks for TKA is summarized in Table 2.

**TABLE 2:** Literature summary on peripheral nerve blocks for TKA.

Authors and year	Reference number	Study type	Sample size	Analgesic techniques discussed	Main results
Wang et al. (2025)	[41]	Randomized controlled trial	100	Comparison of ACB before versus after TKA	Administering an ACB before TKA reduces opioid consumption and improves pain relief during hospitalization.
Jaeger et al. (2013)	[45]	Randomized controlled trial	12	ACB versus FNB on quadriceps strength	ACB preserved quadriceps strength and ability to ambulate better than FNB did
Patterson et al. (2015)	[122]	Retrospective chart review	80	ACB versus FNB	ACB provided analgesia comparable to FNB within 24 h and improved physical therapy performance, as demonstrated by greater gait distance
Kuang et al. (2017)	[47]	Meta‐analysis	9 randomized controlled trials (609 patients)	ACB versus FNB	Compared with FNB, ACB shows similar pain control but can better preserve quadriceps muscle strength
Ochroch et al. (2020)	[123]	Randomized controlled trial	119	ACB + IPACK versus ACB	The IPACK block reduced the incidence of posterior knee pain
Li et al. (2020)	[124]	Randomized controlled trial	200	ACB + IPACK versus ACB	ACB + IPACK provided analgesic benefits over ACB alone
Vichainarong et al. (2020)	[125]	Randomized controlled trial	72	ACB + IPACK versus ACB	ACB + IPACK does not reduce the postoperative opioid consumption nor improve analgesia
Hussain et al. (2021)	[56]	Meta‐analysis	9 trials (1044 patients)	ACB + IPACK versus ACB	When combined with LIA, adding IPACK to ACB provides no additional benefit; without LIA, it reduces pain and improves function.
Hussain et al. (2023)	[57]	Meta‐analysis	11 trials (1185 patients)	Single‐shot ACB versus continuous ACB	Continuous does not enhance or prolong the analgesic benefits compared with single‐shot ACB
Li et al. (2021)	[58]	Retrospective cohort study	530	ACB + additional nerve blocks versus ACB	ACB + additional nerve blocks do not provide superior pain relief compared to ACB when LIA was used

*Note:* IPACK, infiltration between the popliteal artery and capsule of the knee.

Abbreviations: ACB, adductor canal block; FNB, femoral nerve block; LIA, local infiltration analgesia; TKA, total knee arthroplasty.

### 5.2. Peripheral Nerve Blocks for Total Hip Arthroplasty

Multiple peripheral nerve block techniques have been proposed for postoperative analgesia following THA, including quadratus lumborum block, fascia iliaca block, erector spinae block, and pericapsular nerve group (PENG) block [38, 59–63]. However, current high‐quality evidence, including systematic reviews and meta‐analyses, establishes that periarticular LIA remains an important mainstay for analgesia in THA [60, 62–64]. While the combination of motor‐sparing nerve blocks (e.g., PENG block) with LIA may offer incremental benefits in some settings, the evidence for clear and reproducible advantages over LIA alone in key outcomes is inconsistent across studies and centers [65–67]. When administered independently, these peripheral nerve blocks demonstrate no significantly superior analgesia compared to LIA alone [60, 62]. Furthermore, there is no conclusive evidence establishing clear advantages of any specific peripheral nerve block over others [68–72]. We posit that potential contributing factors to the variable evidence may include less severe postoperative pain following THA compared to TKA, the comparative technical nuances and success rates of various peripheral nerve blocks, the adequate analgesic efficacy achieved through optimized intraoperative LIA, and significant heterogeneity in study designs and outcome measures. Therefore, motor‐sparing nerve blocks should be considered optional adjuncts rather than equivalent replacements for LIA, which forms the analgesic mainstay. Consequently, the nursing focus for THA analgesia is often on comprehensive pain assessment and the coordinated administration of systemic multimodal agents, while remaining alert for any signs of block failure or complications if a peripheral nerve block is employed.

The latest key literature on peripheral nerve blocks for THA is summarized in Table 3.

**TABLE 3:** Literature summary on peripheral nerve blocks for THA.

Authors and year	Reference number	Study type	Sample size	Analgesic techniques discussed	Main results
Brixel et al. (2021)	[59]	Randomized controlled trial	100	QLB versus placebo	Adding a posterior QLB to multimodal analgesia reduced neither morphine consumption nor pain scores
Hussain et al. (2022)	[60]	Meta‐analysis	18 trials (1318 patients)	QLB versus control	Do not support the routine use of QLB as part of multimodal analgesic regimens for THA
Gao et al. (2019)	[61]	Meta‐analysis	7 trials (325 patients)	FIB versus placebo	FIB was effective for pain relief during the early postoperative period
Safa et al. (2024)	[62]	Randomized controlled trial	134	FIB versus placebo	FIB did not demonstrate any advantages
Kukreja et al. (2023)	[38]	Randomized controlled trial	112	PENG block versus no block	Adding a PENG block to a multimodal analgesia regimen improves the quality of recovery and reduces opioid requirements
Hu et al. (2023)	[126]	Randomized controlled trial	90	PENG block + LIA versus LIA	PENG block combined with LIA could improve postoperative pain relief, reduce opioid use, and enhance recovery
Hu et al. (2021)	[66]	Randomized controlled trial	80	QLB + LIA versus LIA	QLB combined with LIA can provide better postoperative pain relief and enhance the recovery
Wang et al. (2022)	[127]	Randomized controlled trial	100	QLB versus FIB	FIB provided similar pain relief compared with QLB, but was associated with muscle weakness within 6 h after surgery
Hashmi et al. (2022)	[71]	Randomized controlled trial	50	QLB versus FIB	FIB provided similar pain relief compared with QLB
Et et al. (2023)	[72]	Randomized controlled trial	89	PENG block versus QLB versus LIA	PENG and QLB groups showed similar analgesic effects and both better than the LIA group

*Note:* Results for PENG block and QLB vary across studies and centers. Although some studies have reported improved postoperative analgesia, current evidence remains heterogeneous, and LIA continues to be regarded as the analgesic mainstay for total hip arthroplasty according to current evidence and guideline recommendations. PENG, pericapsular nerve group block.

Abbreviations: FIB, fascia iliaca block; h, hour(s); LIA, local infiltration analgesia; QLB, quadratus lumborum block; THA, total hip arthroplasty.

## 6. Intraoperative Multimodal Analgesia

Current intraoperative analgesic approaches for TJA within ERAS pathways encompass a multimodal strategy, primarily involving intravenous opioids, inhaled anesthetics, intravenous corticosteroids, and periarticular LIA [73]. The selection and administration of these agents are tailored to surgical complexity and patient factors, with a primary goal of facilitating rapid emergence, effective pain control, and seamless transition to postoperative recovery protocols.

### 6.1. Intravenous Opioids During TJA

During TJA, anesthesiologists administer intravenous opioids based on the patient’s vital signs to manage pain from noxious stimuli (e.g., incision, osteotomy) [73]. For the nursing and multidisciplinary team, understanding the distinct roles and typical clinical contexts for different opioids is crucial for anticipatory care, safe handover, and effective postoperative analgesic bridging. Key considerations for clinical workflow integration include the following: (1) Pre‐emptive Communication: Clear preoperative handover regarding a patient’s opioid tolerance history is essential for the anesthesia team to tailor intraoperative dosing and for the postoperative team to anticipate analgesic needs. (2) Monitoring and Transition: Nursing vigilance in the postanesthesia care unit (PACU) is critical, especially following ultra‐short‐acting agents, to ensure timely initiation of multimodal analgesia and prevent pain exacerbation. For patients receiving longer‐acting opioids, nurses must monitor for delayed respiratory depression. (3) Multidisciplinary Planning: The choice between opioid types often involves collaborative decision‐making between the surgeon, anesthesiologist, and pain management team, factoring in discharge timelines and outpatient support.

A summary of commonly used intravenous opioids, their primary intraoperative roles, and key implications for nursing and team‐based care is provided in Table 4.

**TABLE 4:** Overview of common intravenous opioids in TJA: clinical use and implications for care.

Agent	Reference number	Clinical role and context	Implications for perioperative team
Fentanyl	[115]	Frequently used for induction, supplementation, and bolus dosing before intense surgical stimuli (e.g., bone cutting, implant insertion).	Rapid onset but short duration. Requires repeated boluses or infusion for sustained effect. Postoperative monitoring for rapid offset of analgesia is important.
Sufentanil	[116]	Preferred for complex or prolonged procedures requiring high‐potency, sustained analgesia. Often used for both induction and maintenance.	High potency and longer duration. Requires extended postoperative monitoring for respiratory depression. Facilitates stable anesthesia in hemodynamically challenging cases.
Remifentanil	[117]	Ideal for fast‐track anesthesia (e.g., same‐day discharge) and hemodynamically unstable patients due to its precise controllability.	Ultra‐short acting; analgesia ceases minutes after stopping infusion. Mandates proactive postoperative analgesia before discontinuation to avoid severe rebound pain. Typically administered via target‐controlled infusion (TCI).
Morphine	[118]	Not typically a first‐line agent for intraoperative maintenance due to its pharmacokinetic profile.	Slow onset and long duration with active metabolites. A low dose may be given pre‐incision for early postoperative effect. Primary role is in postoperative pain management; nurses should be aware if administered intraoperatively for extended monitoring.

Abbreviations: e.g., for example; TCI, target‐controlled infusion; TJA, total joint arthroplasty.

### 6.2. Inhaled Anesthetics During TJA

Within the ERAS framework for TJA, inhaled anesthetics are a key component of balanced anesthesia, working synergistically with intravenous opioids. While their specific administration is managed by the anesthesia team, understanding their overall impact on patient recovery is crucial for the entire perioperative team, including nursing, to ensure coordinated care and smooth postoperative transitions [73].

The selection of a specific inhaled anesthetic is a strategic decision made by the anesthesiologist, directly influencing the patient’s pathway through the ERAS protocol. This choice affects emergence time, immediate postoperative neurological status, and the timing for key nursing assessments and mobilization goals. Consequently, proactive communication between the anesthesia and nursing teams regarding the planned agent is vital for setting appropriate postoperative expectations and aligning care plans.

A summary of common inhaled anesthetics, their strategic use in different surgical scenarios, and the implications for the nursing and clinical team is provided in Table 5.

**TABLE 5:** Overview of inhaled anesthetics in TJA: strategic use and team implications.

Agent	Reference number	Clinical role and context	Implications for perioperative team
Sevoflurane	[119]	The most common agent for general anesthesia in TJA. Favored for its overall clinical versatility and hemodynamic stability.	Suitable for mask induction and easy titration during maintenance. Its rapid emergence profile facilitates timely neurological assessment in PACU and supports early physical therapy mobilization goals.
Desflurane	[120, 121]	First‐line choice for fast‐track and outpatient TJA protocols where ultra‐rapid emergence is a priority to meet accelerated discharge goals.	Allows for extremely rapid emergence and extubation. Nursing Note: Its airway irritant properties necessitate careful monitoring for respiratory events (e.g., coughing, laryngospasm) upon emergence.
Isoflurane	[121]	Less commonly used as a primary agent in modern TJA practice due to pharmacological profile.	Slower emergence compared to sevoflurane and desflurane. Its more pronounced vasodilatory effects may require more vigilant blood pressure management intra‐ and postoperatively.

Abbreviations: e.g., for example; PACU, postanesthesia care unit; TJA, total joint arthroplasty.

### 6.3. Intravenous Glucocorticoids During TJA

Intravenous corticosteroids are now widely utilized for postoperative pain management in TJA [74, 75]. The latest guidelines from the American Association of Hip and Knee Surgeons (AAHKS) indicate that intraoperative intravenous dexamethasone reduces postoperative pain, mitigates inflammatory responses, and significantly enhances knee functional outcomes following TJA [76]. Compared to a single intraoperative dose, perioperative multidose dexamethasone further alleviates early postoperative pain and decreases opioid consumption [74, 76]. This reduction in opioid use directly translates to a decreased nursing workload in monitoring for and managing opioid‐related adverse effects, such as sedation and respiratory depression. To date, no gold standard exists for dexamethasone dosing in TJA patients. Significant variability persists in reported dexamethasone regimens across studies, with intravenous doses ranging from 4 mg to 20 mg [76]. This variability underscores the nursing imperative to verify the prescribed dose and indication before administration, and to clearly document the administered dose in the medication record to inform ongoing care. This wide dosing range may substantially impact both therapeutic efficacy and complication risks, necessitating further research to establish the optimal dexamethasone dose that maximizes clinical benefits while minimizing associated risks. Furthermore, the ideal timing and duration of dexamethasone therapy require clarification.

Nursing Management of Risks and Contraindications:

Contraindications and risks specific to TJA remain undefined, placing a critical emphasis on vigilant nursing assessment and monitoring:

1. Diabetes: The use of dexamethasone requires caution in diabetic patients. This mandates a thorough nursing review of the patient’s history before administration and collaboration with the physician team for any diabetic patients. Subsequently, close monitoring of blood glucose levels is essential in the postoperative period, typically for 24–72 h depending on the dose and patient response.
2. Other Uncertain Risks: Potential associations with glucocorticoid‐induced osteonecrosis of the femoral head and adrenal‐related complications lack conclusive evidence [76]. Nursing vigilance in assessing and reporting any new or worsening pain (especially hip pain) or unexpected fatigue remains the primary safeguard against these potential yet ill‐defined risks.

### 6.4. LIA

LIA is the cornerstone of intraoperative multimodal analgesia for TJA and serves as a critical foundation for the nursing management of early postoperative pain. LIA targets local sensory nerve endings by injecting a multi‐ingredient cocktail (typically local anesthetics combined with various adjuvants) at multiple sites throughout the surgical field [77]. Critically, LIA preserves muscle strength and does not impede functional recovery [77], which directly supports the nursing and physiotherapy goals of facilitating early and safe ambulation. Consequently, it remains the core intraoperative analgesic technique in contemporary TJA practice.

The specific components of LIA cocktails vary, but nursing awareness of the common agents used is essential for anticipating the duration of action and monitoring for potential side effects (e.g., monitoring for local anesthetic systemic toxicity signs with high doses of ropivacaine/bupivacaine, or glycemic changes with adjuvants, such as dexamethasone). No gold standard exists for the components of LIA cocktails, which typically comprise long‐acting local anesthetics (e.g., ropivacaine, bupivacaine) as primary agents and adjuvants including opioids, NSAIDs, glucocorticoids, epinephrine, and magnesium sulfate [78–80]. The key findings on adjuvant efficacy from a recent systematic review and network meta‐analysis of 43 randomized controlled trials, which are vital for informing nursing expectations of analgesic duration and opioid‐sparing effects, are summarized in Table 6 [81].

**TABLE 6:** Analgesic outcomes of various adjuvants: A summary of a systematic review and network meta‐analysis.

Adjuvant combination	Duration of analgesia (mean ratio [95% CI])	24‐h rest pain score change (mean difference [95% CI])	24‐h morphine consumption (consumption ratio [95% CI])
Number of studies and patients	30 studies, 1866 patients	34 studies, 2157 patients	21 studies, 1284 patients
Clonidine + morphine	3.35 [1.82–6.17]	−1.49 [−2.30–−0.67]	/
Dexamethasone	3.33 [2.24–4.94]	−0.52 [−0.96–−0.08]	/
Morphine + magnesium sulfate	2.92 [1.58–5.40]	−0.90 [−1.73–−0.07]	/
Fentanyl	2.27 [1.44–3.59]	/	/
Ketorolac	2.26 [1.39–3.66]	−1.13 [−1.82–−0.52]	/
Buprenorphine	2.04 [1.20–3.47]	/	/
Morphine	1.93 [1.42–2.28]	−0.62 [−1.04–−0.20]	/
Magnesium sulfate	1.91 [1.30–2.81]	/	/
Clonidine	1.89 [1.33–2.70]	/	0.50 [0.25–0.98]
Dexmedetomidine	1.74 [1.42–2.14]	−0.50 [−0.77–−0.23]	0.57 [0.46–0.72]
Tramadol	1.58 [1.09–2.31]	−1.08 [−1.84–−0.31]	/

*Note:* “/” indicates not reported or not applicable.

Abbreviation: CI, confidence interval.

From a nursing management perspective, the primary practical implications of LIA are as follows: (1) Establishing Expectations: Understanding that the LIA provides a foundational block of analgesia, typically lasting 6–18 h depending on the cocktail used, which establishes a critical window for initiating aggressive physical therapy and multimodal oral analgesia before its effects fully recede [81]. (2) Monitoring Efficacy: Vigilant pain assessment is required as the LIA block wears off to anticipate and proactively address the potential for rebound pain, ensuring a smooth transition to reliance on systemic analgesics. (3) Coordinating Care: Communicating with the surgical team to confirm the specific LIA agents used can directly inform and refine the nursing plan of care for pain management and monitoring.

Recent randomized controlled trials report that novel cocktail formulations (ropivacaine + epinephrine + dexamethasone + magnesium sulfate + sodium bicarbonate) significantly enhance postoperative analgesia, prolong analgesic duration, reduce opioid consumption, and lower VAS pain scores compared to conventional cocktails (ropivacaine + epinephrine + dexamethasone) in both TKA and THA [78, 82]. This evolution directly benefits nursing by simplifying pain management and reducing the workload associated with managing opioid‐related adverse effects. While diverse adjuvants have been documented—some demonstrably extending analgesia duration and enhancing efficacy—the optimal drug combination and dosing regimen for LIA cocktails remain undefined and require further investigation.

## 7. Postoperative Multimodal Analgesia

Postoperative multimodal analgesia protocols for TJA encompass oral analgesics, injectable analgesics, patient‐controlled opioid analgesia, epidural analgesia, cryotherapy, among others. Physical therapies, such as cryotherapy and elevation of the affected limb, serve only as adjunctive analgesic approaches [83]. Patient‐controlled opioid analgesia and epidural analgesia represent traditionally well‐established postoperative analgesic techniques. However, substantial clinical evidence indicates their significantly higher rates of adverse effects, limiting utility within ERAS protocols [42, 84–86]. Patient‐controlled opioid analgesia increases the incidence of postoperative nausea/vomiting, respiratory depression, and urinary retention [84], while epidural analgesia is associated with hypotension, epidural hematoma, and postdural puncture headache [85, 86]. Consequently, both modalities have been largely discontinued in contemporary ERAS‐based multimodal analgesia pathways for TJA.

The core strategy of postoperative multimodal analgesia for TJA involves combining oral and injectable analgesics with distinct mechanisms to target multiple pain pathways, achieving synergistic effects while minimizing doses and adverse reactions—particularly opioid‐related ones [41]. As previously mentioned, for patients experiencing severe pain immediately postoperatively in the PACU, anesthesiologists typically administer intravenous sufentanil. This highly lipophilic opioid provides potent, long‐lasting analgesia (several hours), rapidly controlling acute pain and facilitating transition to ward‐based analgesia [41]. Nursing collaboration in the PACU is critical for assessing pain before administration and ensuring a smooth handoff to the ward team, communicating the analgesic plan and establishing a timeline for reassessment as the sufentanil effect wanes.

Following safe ward transfer, postoperative pain management relies on systematic non‐opioid‐based protocols supplemented with as‐needed opioids. This transition marks the shift to nursing‐led pain management, requiring vigilant assessment, scheduled administration of first‐line agents, and meticulous documentation. Current ERAS and the AAHKS guidelines recommend NSAIDs (including COX‐2 inhibitors) and acetaminophen as first‐line, opioid‐sparing analgesics for post‐TJA pain control, primarily in low‐risk patients [87, 88]. However, this recommendation requires careful risk stratification. Nursing and clinical teams must be aware that NSAIDs require caution and are often contraindicated in elderly individuals, and in patients with renal dysfunction, peptic ulcer disease, or increased bleeding risk. Emphasizing meticulous patient selection and implementing multidisciplinary monitoring are essential for safe administration [89, 90].

In practice, oral NSAIDs typically employ highly selective COX‐2 inhibitors (e.g., celecoxib), which provide excellent anti‐inflammatory analgesia while minimizing platelet inhibition and gastrointestinal complications. Injectable NSAIDs include the highly selective COX‐2 inhibitor parecoxib sodium (requiring IV/IM administration, rapidly hydrolyzed to active valdecoxib) or the nonselective but formulation‐optimized flurbiprofen axetil (delivered via lipid microspheres for targeted accumulation at inflammatory sites, reducing systemic side effects), offering potent nonopioid alternatives for early postoperative periods or patients with oral restrictions. The nursing responsibility encompasses verifying the route and indication for these injectable agents, administering them per protocol, and monitoring for both therapeutic effect and potential adverse reactions.

A structured, nursing‐coordinated approach is imperative when the foundational nonopioid regimen is insufficient: When pain control remains inadequate under this foundational regimen, extended‐release oral opioids (e.g., oxycodone hydrochloride ER and tramadol hydrochloride ER) are recommended as first‐tier rescue analgesics, maintaining stable plasma concentrations for background analgesia [91]. Nursing management at this tier involves patient education on the purpose of scheduled versus as‐needed medications, strict adherence to scheduled dosing to prevent pain breakthrough, and ongoing assessment of sedation and respiratory status. Pain should be reassessed after an appropriate interval following oral rescue analgesia according to institutional protocols. If clinically significant pain persists (e.g., pain score > 4/10 despite first‐tier rescue therapy), escalation to second‐tier rescue analgesia may be considered after multidisciplinary clinical assessment. Should first‐tier oral rescue prove insufficient, second‐tier rescue initiates using injectable potent opioids (e.g., morphine hydrochloride injection) for breakthrough pain management, though strict cardiorespiratory monitoring is mandatory due to risks, such as respiratory depression [92]. This escalation necessitates a validated nursing pain assessment, clear communication with the prescribing physician, and often, dual‐nurse verification of the dose. Continuous monitoring of oxygen saturation and respiratory rate is essential following administration, with clear parameters for escalation of care.

Ultimately, effective postoperative analgesia is a dynamic process requiring seamless interprofessional collaboration. Nurses serve as the coordinators, constantly evaluating the patient’s response, communicating with the surgical and anesthesia teams, and adjusting the plan of care within established protocols to ensure both optimal pain relief and patient safety [93].

## 8. Other Adjuvant Medications

The following adjunctive medications may be incorporated into multimodal regimens. Nursing awareness of their specific profiles is crucial for safe administration and monitoring.

### 8.1. Nefopam

Nefopam is a novel centrally acting analgesic agent that is nonopioid and non‐NSAID. Its analgesic mechanism is unique and not yet fully elucidated, but current research suggests synergistic actions primarily through the following pathways: modulating monoamine neurotransmitters, antagonizing NMDA receptors, and inhibiting voltage‐gated calcium channels [12]. Nefopam does not bind to opioid receptors, thereby lacking typical opioid‐associated side effects, such as respiratory depression, addiction, and constipation. It also does not inhibit COX enzymes, eliminating risks of gastrointestinal ulcers, bleeding, or effects on platelet function, while exhibiting minimal impact on renal function. However, nurses should monitor for its own unique side effects, including nausea, vomiting, sweating, and occasional tachycardia [94]. Recent studies indicate that oral or intravenous administration of nefopam after TKA provides significant postoperative analgesic efficacy [12, 95]. Some researchers propose nefopam as a promising analgesic agent and recommend its incorporation into routine multimodal analgesic regimens following TJA [96, 97].

### 8.2. Antianxiety Medications

Duloxetine is a serotonin and norepinephrine reuptake inhibitor with antidepressant, anxiolytic, and centrally acting analgesic effects. It has been approved for chronic pain management (e.g., diabetic peripheral neuropathy, and fibromyalgia). A recent meta‐analysis incorporating 11 studies involving 1019 patients demonstrated that perioperative administration of duloxetine reduces pain within a span of 3 days–8 weeks after TKA and decreases opioid consumption within 24 h postoperatively [98]. Additionally, it improves knee range of motion and emotional functioning (e.g., depression, anxiety) in patients during the 1–6 weeks following surgery. Researchers identify unexplained postoperative pain as one of the most dreaded complications after TKA. Persistent nociceptive peripheral stimulation can induce central sensitization, wherein the central nervous system becomes hyperexcitable, leading to hypersensitivity to both noxious and non‐noxious stimuli. Patients with central sensitization may be more susceptible to unexplained pain after TKA [34]. This highlights a key nursing role in identifying patients who might benefit from such adjuncts, often in collaboration with pain specialists. In the study, the Central Sensitization Inventory was used to screen for central sensitization in TKA patients preoperatively. Among 464 patients scheduled for primary unilateral TKA due to primary osteoarthritis, 80 were identified with central sensitization and enrolled. These patients were randomized into a duloxetine group (duloxetine 30 mg administered from 1 day preoperatively to 6 weeks postoperatively) and a control group (no duloxetine). Results showed that the duloxetine group exhibited superior outcomes in all pain metrics from 2 to 12 weeks postoperatively. Emotional and physical functioning at 2 weeks postoperatively were also better in the duloxetine group, with no intergroup differences in adverse event rates [34]. Thus, perioperative duloxetine may be considered for central sensitization patients to alleviate postoperative pain. Nursing care for these patients includes monitoring for mood improvement and assessing pain characteristics to evaluate treatment response.

### 8.3. Gabapentinoids

Based on current high‐quality evidence, gabapentinoids (pregabalin and gabapentin) serve as optional adjuncts for analgesia in the multimodal management of pain following TJA, but are not strongly recommended as a routine component for all patients [33]. The collective clinical practice guidelines indicate that while perioperative pregabalin can reduce opioid consumption, its effect on postoperative pain is inconsistent, and gabapentin shows limited efficacy for either outcome. Significant safety concerns exist, particularly regarding an increased risk of sedation and potential respiratory depression when co‐administered with opioids, necessitating extreme caution in the elderly. When gabapentinoids are co‐administered with opioids, regular sedation assessment using validated tools, such as the Pasero Opioid‐Induced Sedation Scale (POSS), together with respiratory monitoring, should be incorporated into routine nursing surveillance. Furthermore, evidence is lacking to define the optimal dose, duration, and direct comparative efficacy between these agents. Therefore, their use should be considered on a selective, individualized basis rather than as a standard recommendation, prioritizing the lowest effective dose to mitigate risks when employed.

### 8.4. Sedative‐Hypnotic Agents

There is no high‐quality evidence supporting a central role for sedative‐hypnotic medications (e.g., benzodiazepines, zolpidem) in the core management of post‐TJA pain. Postoperative pain management primarily relies on the aforementioned multimodal analgesic regimen. Some studies explore the adjunctive role of sedative‐hypnotics, mainly targeting patients with preoperative anxiety or sleep disorders, as these agents may indirectly influence pain perception by improving psychological states [99–101]. Clinically, vigilance is warranted regarding potential exacerbation of postoperative delirium risk, particularly in elderly patients. This makes nursing assessment for delirium a critical safety checkpoint when these medications are used. Existing guidelines do not list traditional sedative‐hypnotics as routine analgesics for post‐TJA pain. Future clinical practice and research must strictly differentiate between sedative and analgesic effects. Drug selection should comprehensively consider patient age, hepatic/renal function, and cognitive status. Nurses play a key role in advocating for this distinction and assessing whether a patient’s restlessness is due to pain (requiring analgesia) or anxiety/agitation (potentially requiring nonpharmacological interventions first).

### 8.5. Long‐Acting Local Anesthetics

Liposomal bupivacaine (brand name EXPAREL) is an innovative extended‐release local anesthetic formulation that encapsulates conventional bupivacaine within multivesicular lipid particles to achieve sustained drug release. This significantly prolongs analgesia (up to 72 h) and is regarded as a major technological advancement in postoperative pain management, currently commonly used for LIA during TJA [102]. However, its efficacy remains contentious: While some studies indicate that liposomal bupivacaine infiltration can reduce postoperative pain, shorten hospital stays, lower hospitalization costs, and decrease opioid consumption compared to traditional local anesthetics [102], other meta‐analyses demonstrate similar efficacy in postoperative pain control and functional recovery between liposomal and conventional local anesthetics for infiltration [103]. Considering cost‐effectiveness in pain management, researchers argue that liposomal bupivacaine is not recommended as a long‐acting alternative for local infiltration [103]. Another systematic review and meta‐analysis highlights significant heterogeneity across existing studies (e.g., injection techniques, inadequate dose standardization), leading to inconclusive findings regarding its analgesic effect and opioid‐sparing benefits [104]. Specific research in elderly patients (≥ 65 years) further confirmed no significant reduction in opioid consumption with liposomal bupivacaine (93.76 mg vs. 83.72 mg, *p* = 0.569) [105]. Future studies are warranted to investigate its effectiveness in multimodal analgesic regimens for TJA [106]. The primary nursing implication is to be aware of the purported extended duration of action (up to 72 h) to set appropriate patient expectations and to be judicious in adding additional analgesics during this window unless warranted by breakthrough pain.

The latest key literature on adjuvant medications is summarized in Table 7.

**TABLE 7:** Literature summary on adjuvant medications.

Authors and year	Reference number	Study type	Sample size	Surgery type	Analgesic techniques discussed	Main results
Wang et al. (2024)	[12]	Randomized controlled trial	100	TKA	Oral nefopam on multimodal analgesia	Adding oral nefopam to multimodal analgesia resulted in improvements in opioid consumption and pain
Pinsornsak et al. (2022)	[95]	Randomized controlled trial	77	TKA	Continuous nefopam administration	Continuous nefopam administration produced a significant analgesic effect and reduced morphine consumption
Yang et al. (2023)	[98]	Meta‐analysis	11 trials (1019 patients)	TKA	Duloxetine for postoperative recovery	Duloxetine reduced pain during 3 days–8 weeks and lower cumulative opioid consumption within 24 h
Koh et al. (2019)	[34]	Randomized controlled trial	80	TKA	Efficacy of duloxetine in centrally sensitized patients	Tailor duloxetine use in multimodal analgesia to central sensitization severity, preventing persistent pain
Doan et al. (2021)	[100]	Retrospective cohort study	4307	THA and TKA	Preoperative benzodiazepines	Preoperative benzodiazepine use was associated with increased odds of readmission
Wang et al. (2017)	[102]	Meta‐analysis	5 trials (1214 patients)	TKA	Liposomal bupivacaine versus bupivacaine in LIA	Liposomal bupivacaine infiltration promotes superior pain relief and less morphine consumption
Kuang et al. (2017)	[103]	Meta‐analysis	11 trials (96,532 patients)	TKA	Liposomal bupivacaine versus bupivacaine in LIA	Liposomal bupivacaine shows similar pain control and functional recovery
Yayac et al. (2019)	[104]	Meta‐analysis	17 trials	TKA	Liposomal bupivacaine versus LIA or peripheral nerve block	Liposomal bupivacaine may offer a statistically significant benefit, and the increase in pain control may not be clinically significant
Minhaj et al. (2021)	[105]	Retrospective cohort study	287	TKA	Liposomal bupivacaine versus bupivacaine in LIA	No significant advantage for liposomal versus standard bupivacaine.
Hussain et al. (2021)	[106]	Meta‐analysis	17 trials (1836 patients)	TKA	Liposomal bupivacaine versus bupivacaine in LIA	No difference between liposomal and plain bupivacaine for post‐TKA analgesia across pain, opioid use, function, and safety

Abbreviations: h, hour(s); LIA, local infiltration analgesia; THA, total hip arthroplasty; TKA, total knee arthroplasty.

## 9. Implications for Nursing Management and Interprofessional ERAS Practice

Successful implementation of phase‐specific analgesic strategies within ERAS depends on proactive nursing management and effective interprofessional collaboration. Nursing professionals serve as the linchpin in executing, monitoring, and adapting these protocols at the bedside. This section translates the preceding clinical evidence into concrete nursing responsibilities and best practices for optimizing patient outcomes.

### 9.1. Patient Education and Expectation Management

Central to the ERAS pathway is the management of patient expectations through structured, preoperative education led by the nursing team [83, 107]. Education should demystify the “non‐opioid‐first” philosophy, explaining the rationale behind multimodal analgesia for enhancing safety and recovery while minimizing side effects [41]. Nurses must set realistic goals for postoperative pain (e.g., aiming for a tolerable pain level of ≤ 4/10 to facilitate mobilization, rather than complete absence of pain), consistent with ERAS principles emphasizing functional recovery and early mobilization [108]. Ideally initiated in the pre‐admission clinic, this education empowers patients to participate actively in recovery and may reduce anxiety‐related pain amplification [107, 109].

### 9.2. Pain Assessment, Mobilization, and Adverse Event Monitoring

Nurses are responsible for the continuous cycle of assessing pain and response to interventions. This extends beyond routine pain scoring using standardized tools (e.g., NRS/VAS) [110]. It requires interpretive assessment: differentiating between incisional pain, neuropathic pain, or pain from muscle spasm, as each may necessitate a different intervention [111].

Mobilization: Nurses coordinate closely with physiotherapists to ensure analgesia is peaking before scheduled mobilization sessions, assessing the patient’s readiness and safety for ambulation.

Systematic Adverse Event Monitoring: Vigilant monitoring for medication‐specific side effects is a paramount nursing duty: (1) NSAIDs/COX‐2 Inhibitors: Nurses should monitor for signs of gastrointestinal intolerance, assess renal function (e.g., intake/output balance), and observe for any unusual bleeding or bruising, especially in at‐risk populations [89, 90]. (2) Duloxetine: While beneficial for patients with central sensitization [34], its use requires nursing awareness of potential neuropsychiatric effects (e.g., nausea, dizziness, somnolence, or rare mood changes) and support for patients during the initial dosing period. (3) Nefopam: Nurses should be aware that while it lacks opioid‐like respiratory depression, common side effects, such as nausea, sweating, or tachycardia, may occur and should be managed symptomatically [96, 97]. (4) Opioids: Even with a reduced reliance, monitoring for respiratory depression (especially with parenteral rescue doses), sedation, nausea, and constipation remains a critical nursing function, utilizing tools, such as the POSS [112].

### 9.3. Nursing Roles in Key Clinical Procedures

Postoperative Opioid Titration: Nursing management is central to the “on‐demand step‐up” rescue strategy. This involves safely administering prescribed oral extended‐release opioids for background pain, assessing their effect, and making informed decisions about the necessity for secondary rescue with intravenous opioids. This requires excellent clinical judgment and strict adherence to monitoring protocols for respiratory status and sedation levels when potent opioids are used [92].

Regional Catheter Management: For patients with continuous peripheral nerve blocks, nursing responsibilities include meticulous assessment of catheter insertion sites for signs of infection or dislodgement, monitoring the infusion pump function, and ongoing assessment of both sensory block and motor function to ensure safety and detect any complications early [57].

Assessing Discharge Readiness: Nurses are pivotal in determining when a patient meets criteria for safe discharge. Key indicators include adequate pain control on a stable, oral multimodal regimen (typically ≤ 4/10 on movement), ability to ambulate a predetermined distance, independent performance of activities of daily living, absence of unmanaged side effects (e.g., nausea, dizziness), and both the patient’s and family’s confidence in managing recovery at home. This comprehensive assessment ensures that the benefits of ERAS are not undone by premature discharge or preventable readmissions [113].

### 9.4. Resource Considerations and Implementation Feasibility

The implementation of contemporary multimodal analgesia pathways may be influenced by institutional resources, workforce capacity, and local expertise. From a nursing management and health service planning perspective, successful adoption of ERAS analgesia protocols requires consideration of both clinical effectiveness and operational feasibility.

Access to advanced regional anesthesia techniques may vary substantially across healthcare settings. Ultrasound‐guided procedures, such as ACB, IPACK block, and PENG block, require specialized equipment, trained anesthesiology personnel, and ongoing competency maintenance. Consequently, some institutions may be unable to routinely provide these techniques despite their demonstrated clinical benefits. In such circumstances, nursing managers and multidisciplinary teams may need to prioritize alternative evidence‐based strategies, including optimized LIA and systemic multimodal analgesia protocols.

Similarly, the use of continuous peripheral nerve catheters introduces additional staffing and monitoring requirements. Effective catheter management requires nursing personnel who are trained in catheter‐site assessment, infusion pump management, neurological monitoring, and complication recognition. Healthcare organizations with limited staffing resources may find single‐injection nerve block techniques more feasible than continuous catheter‐based approaches, particularly given the current evidence suggesting limited additional benefit of routine continuous ACB compared with single‐injection techniques.

Nursing leaders play a critical role in adapting ERAS analgesia pathways to local resource availability while maintaining patient safety and quality of care. This includes workforce planning, competency‐based education, development of standardized protocols, and ongoing evaluation of clinical outcomes. Flexible implementation strategies that balance evidence‐based recommendations with organizational capabilities may facilitate broader adoption of multimodal analgesia while minimizing unwarranted variation in practice.

### 9.5. Fostering Interprofessional Collaboration

The complexity of modern personalized multimodal analgesia demands seamless interprofessional communication [93]. Nursing professionals act as the central communication hub, facilitating collaboration between surgeons, anesthesiologists, pain specialists, physiotherapists, and pharmacists. This includes the following: (1) Preoperative: Communicating a patient’s risk stratification (e.g., opioid tolerance, central sensitization score, renal impairment) to the entire team to tailor the perioperative plan. (2) Intraoperative: Receiving a clear handover from the anesthesia team regarding analgesics administered, regional techniques performed, and any specific postoperative monitoring requirements. (3) Postoperative: Leading regular interdisciplinary rounds to discuss patient progress, report on pain scores and opioid consumption, address barriers to mobilization, and adjust the care plan in real‐time based on the patient’s response.

In conclusion, the transition toward personalized multimodal analgesia elevates the role of nursing from protocol administrators to essential clinical decision‐makers and coordinators within the ERAS team [114]. Their expertise in patient education, vigilant monitoring, and collaborative practice is indispensable for translating advanced analgesic evidence into superior, safe, and personalized patient outcomes.

## 10. Practical Implementation Tools for Nursing Management and Interprofessional ERAS Analgesia

### 10.1. Phase‐Based Nursing Checklist for Pain Management and Monitoring Priorities

To facilitate translation of evidence into routine clinical practice, a phase‐based nursing checklist is proposed (Table 8). The checklist summarizes key nursing responsibilities, monitoring priorities, medication‐specific safety considerations, and escalation criteria across the preoperative, intraoperative, and postoperative phases of TJA. Particular emphasis is placed on monitoring high‐risk analgesic therapies, including NSAIDs, gabapentinoids administered in combination with opioids, regional analgesic techniques, and opioid rescue therapy. The tool is intended to support protocol standardization, staff education, quality assurance, and consistent implementation of ERAS‐based multimodal analgesia pathways.

**TABLE 8:** Phase‐based nursing checklist for perioperative multimodal analgesia in total joint arthroplasty.

Phase	Nursing priority	Key nursing actions	Monitoring focus
Preoperative	Risk stratification and patient preparation	Verify medication history; identify opioid tolerance; screen for central sensitization; assess renal function, diabetes, bleeding risk; reinforce ERAS education and pain expectations	Baseline pain score; medication contraindications; anxiety level; fall risk
Preoperative	Preemptive multimodal analgesia	Administer prescribed preoperative medications (e.g., COX‐2 inhibitors, pregabalin, duloxetine where indicated); verify timing of administration	Dizziness, somnolence, gastrointestinal symptoms, allergic reactions
Preoperative	Patient education	Explain multimodal analgesia strategy, realistic pain goals, early mobilization expectations, and rescue analgesia pathway	Patient understanding and readiness
Intraoperative	Communication and continuity of care	Confirm completion of peripheral nerve blocks and LIA; document intraoperative dexamethasone and opioid administration; prepare postoperative monitoring plan	Block type; dexamethasone dose; intraoperative opioid exposure
Intraoperative	Handover preparation	Receive structured anesthesia handover before PACU/ward transfer	Analgesic plan; expected block duration; anticipated rebound pain window
Postoperative (0–24 h)	Early pain assessment	Assess pain at rest and during movement using NRS/VAS; reassess after analgesic interventions	Pain trajectory; breakthrough pain
Postoperative (0–24 h)	Nonopioid‐first analgesia	Administer scheduled NSAIDs/COX‐2 inhibitors and acetaminophen according to protocol; verify contraindications in elderly patients and those with renal impairment, peptic ulcer disease, or bleeding risk	Gastrointestinal intolerance; bleeding; renal function; high‐risk patient screening
Postoperative (0–24 h)	Regional block monitoring	Evaluate sensory and motor function; assess effectiveness of ACB/IPACK/LIA; monitor for block‐related complications	Motor weakness; numbness; falls risk
Postoperative (0–72 h)	Liposomal bupivacaine monitoring	Recognize that severe pain occurring within the expected analgesic duration may represent breakthrough pain rather than immediate block failure; continue multimodal analgesia and reassess. Escalate evaluation if persistent dense motor block exceeds expected duration	Breakthrough pain; persistent motor blockade (> 48 h); neurologic reassessment
Postoperative (0–48 h)	Mobilization support	Coordinate analgesic peak effect with physiotherapy sessions; evaluate readiness for ambulation	Mobility tolerance; dizziness; orthostatic symptoms
Postoperative (early postoperative period)	Gabapentinoid‐opioid co‐administration monitoring	Assess sedation using the Pasero Opioid‐Induced Sedation Scale (POSS) at regular intervals during concurrent gabapentinoid‐opioid therapy	POSS score; respiratory rate; oxygen saturation; notify treating team if POSS ≥ 3 or respiratory rate < 10 breaths/min
Postoperative (as needed)	Opioid rescue management	Reassess pain after first‐tier rescue analgesia according to institutional protocol; consider escalation when clinically significant pain persists despite initial rescue therapy	Pain score; respiratory rate; oxygen saturation; sedation score
Postoperative (through discharge)	Adverse event surveillance	Monitor for opioid‐related adverse effects, NSAID‐related complications, duloxetine‐associated symptoms, and dexamethasone‐related hyperglycemia	Sedation, respiratory depression, nausea, constipation, glucose control
Discharge	Readiness assessment	Confirm pain control on oral regimen, functional mobility, patient confidence, and understanding of home analgesia plan	Pain ≤ 4/10 during mobilization; medication adherence; safety at home

*Note:* COX‐2, cyclooxygenase‐2; IPACK, infiltration between the popliteal artery and capsule of the knee; POSS, Pasero Opioid‐Induced Sedation Scale.

Abbreviations: ACB, adductor canal block; ERAS, enhanced recovery after surgery; h, hour(s); LIA, local infiltration analgesia; NRS, numeric rating scale; NSAIDs, nonsteroidal anti‐inflammatory drugs; PACU, postanesthesia care unit; VAS, visual analog scale.

### 10.2. Standardized Interprofessional Analgesia Handover Bundle

Effective ERAS implementation requires structured communication between anesthesiology, surgical, and nursing teams. Analgesic techniques administered intraoperatively may substantially influence postoperative monitoring requirements, rehabilitation planning, and patient safety. To reduce communication gaps and support continuity of care, a standardized interprofessional analgesia handover bundle is proposed (Table 9). The bundle focuses on information that directly affects postoperative pain assessment, opioid requirements, mobilization planning, and adverse event surveillance.

**TABLE 9:** Standardized interprofessional analgesia handover bundle for total joint arthroplasty.

Handover domain	Information to be communicated	Nursing implication
Procedure information	TKA or THA; primary or revision procedure	Anticipate expected pain trajectory and rehabilitation requirements
Peripheral nerve blocks	Type of block performed (ACB, IPACK, PENG, fascia iliaca block, etc.)	Guide motor and sensory assessment; identify fall risk
Block timing	Time block was performed	Estimate expected duration of analgesia
Expected rebound pain window	Anticipated time of block resolution	Intensify pain reassessment before analgesic effect diminishes
Local infiltration analgesia	LIA performed; major components if available	Anticipate analgesic duration and transition planning
Intraoperative opioids	Agent(s) administered and approximate cumulative dose	Assess risk of postoperative sedation and respiratory depression
Dexamethasone administration	Dose and timing	Monitor glucose control and document anti‐inflammatory strategy
Special analgesic considerations	Opioid tolerance, chronic pain history, central sensitization, difficult pain control	Individualize pain management plan
Immediate postoperative plan	Scheduled multimodal medications and rescue strategy	Ensure continuity of analgesia
Escalation pathway	Responsible anesthesia/pain management contact	Facilitate rapid response to uncontrolled pain or complications

*Note:* IPACK, infiltration between the popliteal artery and capsule of the knee; PENG, pericapsular nerve group block.

Abbreviations: ACB, adductor canal block; LIA, local infiltration analgesia; THA, total hip arthroplasty; TKA, total knee arthroplasty.

Nursing managers may incorporate this bundle into PACU‐to‐ward transfer forms, electronic health records, or standardized ERAS handover templates to improve communication reliability and reduce variation in postoperative pain management.

### 10.3. Minimum Monitoring Bundle for Opioid Rescue Analgesia

Although contemporary ERAS pathways emphasize opioid‐sparing analgesia, opioid rescue therapy remains necessary for a subset of patients experiencing breakthrough pain. Because opioid administration is associated with potentially serious adverse events, particularly respiratory depression and excessive sedation, a standardized monitoring framework is essential. The following minimum monitoring bundle (Table 10) is proposed to support safe opioid rescue practices and quality assurance across clinical settings.

**TABLE 10:** Minimum monitoring bundle for opioid rescue analgesia.

Monitoring Component	Recommended assessment	Escalation trigger
Pain intensity	NRS/VAS before administration and 30–60 min after intervention	Persistent pain ≥ 7/10 despite rescue therapy
Respiratory rate	Count respirations at regular intervals after opioid administration	RR < 10 breaths/min
Oxygen saturation	Continuous or intermittent pulse oximetry according to institutional protocol	SpO_2_ < 92% or significant decline from baseline
Sedation level	Assess using POSS or institutional sedation scale	Excessive sedation (POSS ≥ 3)
Level of consciousness	Evaluate responsiveness and ability to maintain conversation	Difficult to arouse or unresponsive
Hemodynamic status	Blood pressure and heart rate monitoring	Symptomatic hypotension or hemodynamic instability
Nausea and vomiting	Routine symptom assessment	Persistent symptoms limiting oral intake or mobilization
Mobility and fall risk	Assess before ambulation	New motor weakness, dizziness, or unsteady gait
Bowel function	Monitor constipation in patients receiving repeated opioid doses	No bowel movement with progressive symptoms
Documentation and reassessment	Record intervention, response, and follow‐up assessment	Failure to achieve clinically meaningful pain relief

*Note:* SpO_2_, peripheral oxygen saturation; POSS, Pasero Opioid‐Induced Sedation Scale.

Abbreviations: NRS, numeric rating scale; RR, respiratory rate; VAS, visual analog scale.

For quality assurance purposes, nursing managers may monitor compliance with this bundle through routine audits, documentation reviews, and ERAS performance indicators. Suggested quality metrics include timely pain reassessment, completion of respiratory monitoring after opioid administration, incidence of opioid‐related adverse events, and adherence to escalation protocols.

## 11. Summary and Conclusions

The core objective of perioperative pain management for TJA has shifted from solely pursuing pain relief to achieving a balance between rapid functional recovery and minimized complications within the ERAS framework. Multimodal analgesia, by combining pharmacologic agents with distinct mechanisms and nonpharmacologic techniques, has significantly reduced reliance on traditional opioids and their associated adverse effects. However, current evidence reveals substantial limitations in standardized analgesic protocols: Key aspects, such as drug selection across phases (e.g., medication combinations of preemptive multimodal analgesia, adjuvant formulations for LIA), technical combinations (e.g., types of peripheral nerve blocks), and implementation timing (e.g., timing of dexamethasone administration), lack a unified gold standard. This heterogeneity in evidence underscores a critical nursing management imperative: the development, adoption, and consistent execution of standardized, institution‐specific clinical pathways and analgesic protocols. These protocols are essential tools to bridge evidence gaps, minimize practice variation, and ensure patient safety amidst evolving clinical evidence.

From a nursing management perspective, the limitations of the current evidence base deserve explicit consideration. Although multimodal analgesia is widely accepted as the standard of care for TJA, substantial uncertainty remains regarding several key protocol components. First, no consensus exists on the optimal combination of agents for preemptive multimodal analgesia, with considerable variation in the use of COX‐2 inhibitors, gabapentinoids, acetaminophen, duloxetine, and opioids across studies and institutions. Second, dexamethasone regimens differ markedly with respect to dose, timing, and frequency of administration, making direct comparisons difficult and limiting the development of standardized perioperative pathways. Third, LIA cocktails demonstrate substantial heterogeneity in both constituent agents and dosing strategies, despite widespread clinical adoption. In addition, several recommendations discussed in this review are supported in part by studies conducted by our research group. While these studies contribute valuable evidence to the field and were included based on their methodological quality and relevance, independent external validation remains limited for certain interventions, particularly newer multimodal analgesic strategies and emerging analgesic combinations. Therefore, these findings should be interpreted within the context of the broader available literature, and further multicenter investigations from independent research groups are warranted to confirm their generalizability and strengthen the evidence base.

These evidence gaps have important implications beyond clinical decision‐making. For nursing‐led ERAS programs, the absence of standardized protocols complicates protocol governance, staff education, audit processes, benchmarking across institutions, and continuous quality improvement initiatives. Variability in practice may also contribute to inconsistent patient experiences and difficulties in evaluating the effectiveness of local analgesic pathways. Consequently, nursing managers should adopt evidence‐informed but locally standardized protocols, supported by regular auditing, outcome monitoring, and interdisciplinary review processes. Such governance structures may help reduce unwarranted variation while allowing future protocol refinement as higher‐quality evidence becomes available.

Nevertheless, nursing managers and clinical teams cannot defer protocol implementation while awaiting perfect evidence. Therefore, the best available evidence should be translated into structured, evidence‐informed pathways that can be locally adapted, monitored, and continuously refined. The following recommendations summarize key considerations for implementing personalized multimodal analgesia within contemporary ERAS programs for TJA.

The initiation timing (recommended 24–48 h preoperatively) and drug combinations for preemptive analgesia must be tailored based on the patient’s medication tolerance history, renal function, and central sensitization risk. For instance, high‐risk central sensitization patients (identified through scale‐based screening) are advised to combine duloxetine (30 mg/day starting 1 day preoperatively for 6 weeks) to reduce the incidence of chronic postoperative pain. This screening and subsequent monitoring represent a key nursing role in the pre‐admission testing process, requiring collaboration with surgeons and pain specialists. Conversely, patients with opioid tolerance should avoid extended‐release opioids preoperatively (e.g., oxycodone) and instead intensify nonopioid regimens. For TKA, the combination of ACB, IPACK block, and LIA is strongly supported as the evidence‐based standard regimen to achieve optimal analgesia while preserving quadriceps function and facilitating early ambulation. Nursing’s role is pivotal in coordinating the timing of mobilization with the peak effect of these blocks and in monitoring for motor or sensory deficits. For THA, periarticular LIA is established as the analgesic mainstay, with motor‐sparing nerve blocks (e.g., PENG block) considered optional adjuncts rather than standard replacements, due to inconsistent evidence for their reproducible benefits across centers.

The intraoperative phase requires matching anesthetic strategies to surgical complexity and patient status. For fast‐track procedures, such as same‐day outpatient TJA, a combination of remifentanil and desflurane is administered to achieve rapid emergence. Prolonged complex surgeries favor sufentanil and sevoflurane for sustained analgesia. Dexamethasone (single 8–10 mg intravenous bolus) effectively suppresses inflammatory responses, but diabetic patients require strict dose limitation with glucose monitoring. This mandates clear interdisciplinary communication between the anesthesia and nursing teams regarding intraoperative medications administered to ensure seamless and informed postoperative monitoring. Adjunct selection for LIA cocktails should adhere to evidence‐based optimization principles: prioritize synergistic combinations (e.g., dexamethasone or clonidine + morphine to prolong analgesia duration, or dexmedetomidine for opioid‐sparing effects) while avoiding redundant additions (e.g., tramadol).

The postoperative phase requires constructing a dynamic “non‐opioid‐first, on‐demand step‐up” strategy. First‐line therapy should prioritize NSAIDs (including COX‐2 inhibitors) combined with acetaminophen as opioid‐sparing agents, in alignment with ERAS and AAHKS guidelines. However, this recommendation must be risk‐stratified, applying primarily to low‐risk patients. Caution is required in elderly individuals and those with renal dysfunction, peptic ulcer disease, or increased bleeding risk. Careful patient selection and vigilant multidisciplinary monitoring by the nursing and clinical team are paramount for safe administration. This is operationalized through nursing‐led protocols for scheduled administration, structured pain reassessment, and systematic screening for adverse effects. Should analgesia prove inadequate, rescue protocols are sequentially initiated: Oral extended‐release opioids (e.g., oxycodone) serve as primary rescue, while injectable potent opioids (e.g., morphine) are reserved as secondary rescue only under close monitoring for adverse reactions. Nursing competency in opioid titration—assessing pain, administering doses, and monitoring for efficacy and sedation/respiratory depression—is a cornerstone of safe rescue analgesia. Innovative agents, such as nefopam (a nonopioid centrally acting analgesic), are suitable opioid alternatives for patients with respiratory disorders. Liposomal bupivacaine, due to contentious cost‐effectiveness, is recommended solely for specific individuals with strong opioid‐reduction requirements.

Individualized adjustments for special populations are critical. Elderly patients (≥ 65 years) should avoid sedative‐hypnotics to reduce delirium risk and simplify nerve block management (e.g., single‐shot ACB rather than continuous catheter techniques). Nursing‐driven delirium risk assessment and prevention strategies are non‐negotiable components of care for geriatric patients. Diabetic patients require cautious use of dexamethasone, prioritizing non‐NSAID analgesic bases. Those with opioid tolerance history must completely avoid preoperative opioids, relying instead on nerve blocks and COX‐2 inhibitors. Patients with confirmed central sensitization necessitate integrated duloxetine therapy throughout the entire perioperative period.

Future research should focus on three key domains: First, optimal standards are established for controversial areas (e.g., efficacy‐safety windows for dexamethasone dosing, validation of prophylactic medication combinations). From a nursing management perspective, future studies must also prioritize the implementation science of how to best integrate these evidence‐based protocols into nursing workflows, including the development of decision‐support tools and standardized competencies for pain management. Second, genomics‐based drug response prediction models and dynamic analgesic monitoring technologies are developed. Third, long‐term benefits of personalized regimens are quantified in reducing hospitalization costs and opioid‐related complications. Ultimately, interdisciplinary collaboration (departments of orthopedic surgery, anesthesiology, pain management, and nursing teams) is essential to construct accelerated recovery‐centered precision analgesia pathways, achieving triple optimization of analgesic efficacy, safety, and health economics. This collaboration is most effectively realized through structured interprofessional rounds, shared order sets, and a culture where nursing assessments directly inform analgesic prescribing and adjustments. The success of ERAS pain management depends not only on pharmacological innovation but also on coordinated, nursing‐led interdisciplinary care.

## Author Contributions

Qiuru Wang: conceptualization, literature search, data curation, writing–original draft, and writing–review and editing.

Dongmei Zhao: conceptualization, clinical nursing expertise, writing–review and editing, and validation.

Jian Hu: conceptualization, clinical expertise (anesthesiology), writing–review and editing, and validation.

Changjun Chen: clinical expertise (orthopedic surgery) and writing–review and editing.

Ting Ma: clinical nursing expertise, writing–review and editing, and validation.

Xiaoyan Liu: clinical nursing expertise, supervision of nursing content, writing–review and editing, and validation.

Jing Yang: conceptualization, clinical expertise (anesthesiology), supervision, writing–review and editing, and project administration.

Pengde Kang: conceptualization, supervision, project administration, funding acquisition, and writing–review and editing.

Qiuru Wang, Dongmei Zhao, and Jian Hu contributed equally to this work and should be regarded as co‐first authors.

Pengde Kang and Jing Yang contributed equally as co‐corresponding authors.

## Funding

This study was supported by the General Program of the National Natural Science Foundation of China (Grant No. 82372392) and the Science and Technology Department of Sichuan Province Project (Grant No. 2024NSFSC0568).

## Conflicts of Interest

The authors declare no conflicts of interest.

## Data Availability

Data sharing is not applicable to this article as no datasets were generated or analyzed during the current study.
